# Chromosomal-level genome assembly of golden birdwing *Troides aeacus* (Felder & Felder, 1860)

**DOI:** 10.46471/gigabyte.122

**Published:** 2024-04-25

**Authors:** Hong Kong Biodiversity Genomics Consortium, Jerome H. L. Hui, Ting Fung Chan, Leo Lai Chan, Siu Gin Cheung, Chi Chiu Cheang, James Kar-Hei Fang, Juan Diego Gaitan-Espitia, Stanley Chun Kwan Lau, Yik Hei Sung, Chris Kong Chu Wong, Kevin Yuk-Lap Yip, Yingying Wei, Wai Lok So, Wenyan Nong, Hydrogen Sui Fai Pun, Wing Kwong Yau, Colleen Yuk Lin Chiu, Sammi Shan Shan Chan, Kacy Ka Ling Man, Ho Yin Yip

**Affiliations:** ^1^ School of Life Sciences, Simon F.S. Li Marine Science Laboratory, State Key Laboratory of Agrobiotechnology, Institute of Environment, Energy and Sustainability, The Chinese University of Hong Kong, Hong Kong, China; ^2^ School of Life Sciences, State Key Laboratory of Agrobiotechnology, The Chinese University of Hong Kong, Hong Kong SAR, China; ^3^ State Key Laboratory of Marine Pollution and Department of Biomedical Sciences, City University of Hong Kong, Hong Kong SAR, China; ^4^ State Key Laboratory of Marine Pollution and Department of Chemistry, City University of Hong Kong, Hong Kong SAR, China; ^5^ Department of Science and Environmental Studies, The Education University of Hong Kong, Hong Kong SAR, China; ^6^ EcoEdu PEI, Charlottetown, PE, C1A 4B7, Canada; ^7^ Department of Food Science and Nutrition, Research Institute for Future Food, and State Key Laboratory of Marine Pollution, The Hong Kong Polytechnic University, Hong Kong SAR, China; ^8^ The Swire Institute of Marine Science and School of Biological Sciences, The University of Hong Kong, Hong Kong SAR, China; ^9^ Department of Ocean Science, The Hong Kong University of Science and Technology, Hong Kong SAR, China; ^10^ Science Unit, Lingnan University, Hong Kong SAR, China; ^11^ School of Allied Health Sciences, University of Suffolk, Ipswich, IP4 1QJ, UK; ^12^ Croucher Institute for Environmental Sciences, and Department of Biology, Hong Kong Baptist University, Hong Kong SAR, China; ^13^ Department of Computer Science and Engineering, The Chinese University of Hong Kong, Hong Kong SAR, China; ^14^ Sanford Burnham Prebys Medical Discovery Institute, La Jolla, CA, USA; ^15^ Department of Statistics, The Chinese University of Hong Kong, Hong Kong SAR, China; ^16^ Fung Yuen Butterfly Reserve, Hong Kong SAR, China

## Abstract

The golden birdwing *Troides aeacus* (Lepidoptera, Papilionidae), a significant species in Asia, faces habitat loss due to urbanization and human activities, necessitating its protection. However, the lack of genomic resources hinders our understanding of their biology and diversity, and impedes our conservation efforts based on genetic information or markers. Here, we present the first chromosomal-level genome assembly of *T. aeacus* using PacBio SMRT and Omni-C scaffolding technologies. The assembled genome (351 Mb) contains 98.94% of the sequences anchored to 30 pseudo-molecules. The genome assembly has high sequence continuity with contig length N50 = 11.67 Mb and L50 = 14, and scaffold length N50 = 12.2 Mb and L50 = 13. A total of 24,946 protein-coding genes were predicted, with high BUSCO score completeness (98.8% and 94.7% of genome and proteome BUSCO, respectively. This genome offers a significant resource for understanding the swallowtail butterfly biology and carrying out its conservation.

## Introduction

**Figure 1.**
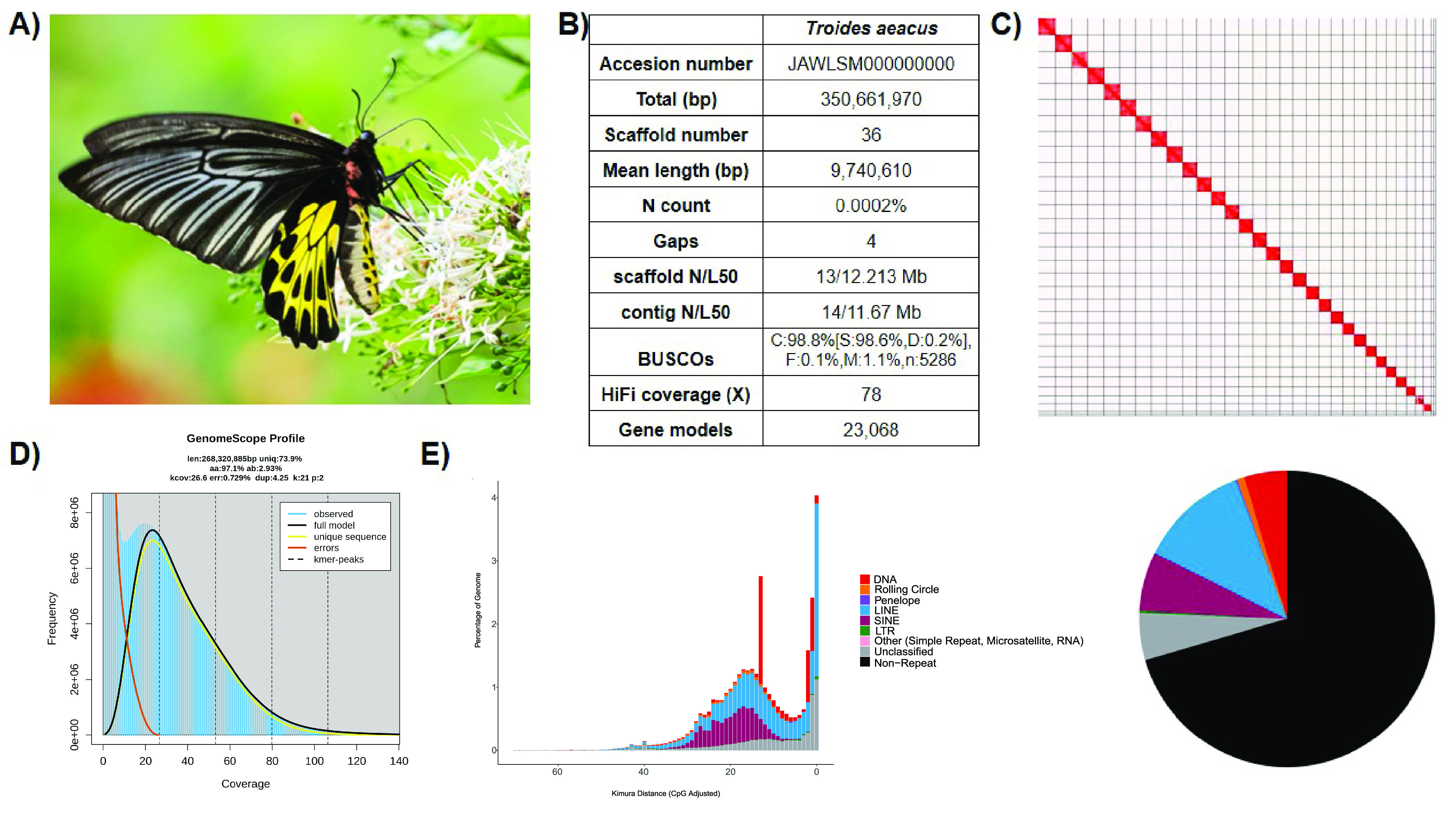
Genomic information of *Troides aeacus*. (A) Photo of *T. aeacus*; (B) Statistics of the assembled genome; (C) Omni-C contact map of the assembly visualised using Juicebox (v1.11.08); (D) Genomescope report with k-mer = 21; (E) Repetitive elements distribution in the assembled genome.

The golden birdwing butterfly *Troides aeacus* (Figure 1A) is a swallowtail butterfly that is widely distributed in Asia, including Bangladesh, Myanmar, Cambodia, China, India, Laos, Malaysia, Nepal, Thailand, and Vietnam [1]. The species is generally large, with a wingspan reaching ∼15 cm, and has iconic black forewings and golden-yellow hindwings carved with grey stripes and black spots [2, 3]. Due to its attractive appearance, it has been vastly collected and traded in curio markets [2, 4, 5].

 Similar to other homometabolans, *T. aeacus* has larvae and pupae stages: five larval instar stages before transforming into its green-girdled pupal stage [3]. The larvae are generally dependent on Aristolochiaceae host plants, especially of the genus *Aristolochia*, which can be commonly found in Asia [1–3, 6]. After emergence, the adults feed and live around nectaring flowers such as those in the genus *Hibiscus*, *Ixora*, *Lantana*, *Mussaenda*, and *Spathodea* [1, 7]. Anthropogenic activities, including deforestation, grazing, herbicide application, hunting, land reclamation, mine exploitation, and trading, have been suggested to pose threats to *T. aeacus* [1, 3, 8]. In certain places, such as Hong Kong, *T. aeacus* has also been suggested for protection and restoration efforts to recover its lost habitat. In Taiwan, the trade of endemic subspecies such as *T. aeacus* is protected by the Convention on International Trade in Endangered Species of Wild Fauna and Flora [9].

## Context

To date, the genomic resources in the genus *Troides* are confined to *T. helena* [10] and *T. oblongomaculatus* [11]. In light of the high conservation value of *T. aeacus* and its phylogenetic importance for understanding the diversification of butterflies [12], this species has been selected for genome sequencing by the Hong Kong Biodiversity Genomics Consortium (also known as EarthBioGenome Project Hong Kong), which is formed by investigators from eight publicly funded universities in Hong Kong. Here, we report a chromosomal-level genome assembly of the golden birdwing *T. aeacus*.

## Methods

### Sample collection and species identification

A pupa of the golden birdwing *T. aeacus* was obtained at Lui Kung Tin, Yuen Long District, Hong Kong (22.425886 °N, 114.10538 °E) in August 2022. The pupa was snap-frozen in liquid nitrogen upon collection. The frozen pupa was then ground into a fine powder and stored at −80 °C until DNA isolation. A portion of the powder was used for species molecular identification with QIAamp DNA Mini Kit (Qiagen Cat. 51306), following the provided protocol. The DNA was then used as a template for conventional PCR with the following protocol: an initial denaturation step at 95 °C for 3 minutes followed by 36 amplification cycles for denaturation of 30 seconds each at 95 °C; 30 seconds for primer annealing at 55 °C and 1 minute for extension at 72 °C; finally, an extension step at 72 °C for 3 minutes. The reaction mixture included PCR buffer, DNA template, 2 mM dNTP, 1.5 mM MgCl_2_, 0.4 mM of each forward and reverse primers (LCO1490: 5′-GGTCAACAAATCATAAAGATATTGG-3′, HCO2198: 5′-TAAACTTCAGGGTGACCAAAAAATCA-3′) [13], and *Taq* DNA polymerase. The PCR was performed on a T100™ thermal cycler (Bio-Rad, USA). The unpurified PCR products were sent to BG Hong Kong for Sanger sequencing. The returned sequence was validated with the chromatogram, and the resultant sequence was searched against Genbank for species validation using the BLASTN algorithm (RRID:SCR_001598).

### Isolation of high molecular weight genomic DNA

High molecular weight genomic DNA was isolated from the remaining stored powder using the Qiagen MagAttract HMW kit (Qiagen Cat. No. 67563) following the manufacturer’s protocol. In summary, 1 g of sample powder was placed in a microcentrifuge tube with 200 μl 1× phosphate-buffered saline (PBS), RNase A, Proteinase K, and Buffer AL. The mixture was incubated at room temperature (22–25 °C) for 2.5 hours until the tissue was completely disintegrated. The sample was then eluted with 120 μl of elution buffer (PacBio Ref. No. 101-633-500) and stored at 4 °C. In order to keep the integrity of the DNA, wide-bore pipette tips were used for any DNA transfer during the process. The sample was then subjected to quality control with the Qubit^®^ Fluorometer, Qubit™ dsDNA HS, and BR Assay Kits (Invitrogen™ Cat. No. Q32851). An overnight pulse-field gel electrophoresis was performed to estimate the size of the isolated DNA using three markers (λ-Hind III digest; Takara Cat. No. 3403, DL15,000 DNA Marker; Takara Cat. No. 3582A and CHEF DNA Size Standard-8-48 kb Ladder; Cat. No. 170-3707). Additionally, the sample purity was examined by the NanoDrop™ One/OneC Microvolume UV–Vis Spectrophotometer (with A260/A280: ∼1.8 and A260/A230: >2.0 as a standard threshold).

### DNA shearing, PacBio library preparation, and sequencing

A total of 120 μl of DNA, corresponding to 10 μg DNA, was transferred to a g-tube (Covaris Part No. 520079). The tube was then subjected to six centrifugation steps with 2,000 × g of 2 minutes each. The resultant DNA was saved in a 2 mL DNA LoBind^®^ Tube (Eppendorf Cat. No. 022431048) at 4 °C until library preparation. The molecular weight of the isolated DNA was examined by overnight pulse-field gel electrophoresis. The electrophoresis profile was set as follows: 5 K as the lower end and 100 K as the higher end for the designated molecular weight; Gradient = 6.0 V/cm; Run time = 15 h:16 min; included angle = 120°; Int. Sw. Tm = 22 s; Fin. Sw. Tm = 0.53 s; Ramping factor: a = Linear. The gel was run in 1.0% PFC agarose in 0.5× TBE buffer at 14 °C.

A SMRTbell library was made using the SMRTbell^®^ prep kit 3.0 (PacBio Ref. No. 102-141-700), following the provided protocol. In summary, single-stranded overhangs of the genomic DNA were removed, and the DNA was repaired from any physical damage caused by shearing. Subsequently, both DNA ends were tailed with an A-overhang, and ligation of T-overhang SMRTbell adapters was performed at 20 °C for 30 minutes. The SMRTbell library was then purified with SMRTbell^®^ cleanup beads (PacBio Ref. No. 102158-300). The size and concentration of the library were assessed with the pulse-field gel electrophoresis and the Qubit^®^ Fluorometer, Qubit™ dsDNA HS, and BR Assay Kits (Invitrogen™ Cat. No. Q32851), respectively. A subsequent nuclease treatment step was carried out to remove non-SMRTbell structures in the library. A final size-selection step was performed to remove small DNA fragments in the library with 35% AMPure PB beads. The Sequel^®^ II binding kit 3.2 (PacBio Ref. No. 102-194-100) was used for final preparation. In short, Sequel II primer 3.2 and Sequel II DNA polymerase 2.2 were annealed and bound to the SMRTbell library, respectively. Next, the library was loaded at an on-plate concentration of 50–90 pM using the diffusion loading mode. The sequencing was conducted on the Sequel IIe System with an internal control provided in the kit. The sequencing was performed in 30-hour movies, with 120 min pre-extension, connected to the software SMRT Link v11.0 (PacBio). HiFi reads were generated and collected for further analysis. One SMRT cell was used for this sequencing (Table 1).

**Table 1:** Summary of the genome sequencing data.

Library	Reads	Bases	Coverage (X)	Accession
PacBio HiFi	2,805,656	27,181,071,888	78	SRR24631717
Omni-C	144,777,842	21,716,676,300	62	SRR26815782

### Omni-C library preparation and sequencing

An Omni-C library was made using the Dovetail^®^ Omni-C^®^ Library Preparation Kit (Dovetail Cat. No. 21005) according to the provided protocol. In summary, 80 mg of frozen, powered tissue sample was placed in a microcentrifuge tube with 1 mL 1× PBS and formaldehyde. The fixed DNA was digested with endonuclease DNase I. Next, the concentration and size of the digested sample were examined by the Qubit^®^ Fluorometer, Qubit™ dsDNA HS, and BR Assay Kits (Invitrogen™ Cat. No. Q32851) and the TapeStation D5000 HS ScreenTape, respectively. Both DNA ends were polished, and ligation of the biotinylated bridge adaptor was conducted at 22 °C for 30 minutes. The subsequent proximity ligation between crosslinked DNA was performed at 22 °C for 1 hour. After ligation, the DNA was reverse crosslinked and purified with SPRIselect™ Beads (Beckman Coulter Product No. B23317) to remove the biotin that was not internal to the ligated fragments. The Dovetail™ Library Module for Illumina (Dovetail Cat. No. 21004) was used for end repair and adapter ligation. The DNA was tailed with an A-overhang, which allowed Illumina-compatible adapters to ligate to the DNA fragments at 20 °C for 15 minutes. The Omni-C library was then sheared into fragments with USER Enzyme Mix and purified with SPRIselect™ Beads. The isolation of DNA fragments with internal biotin was performed with Streptavidin Beads. Universal and Index PCR Primers from the Dovetail™ Primer Set for Illumina (Dovetail Cat. No. 25005) were used to amplify the constructed library. Size selection was carried out with SPRIselect™ Beads targeting fragments ranging between 350 bp and 1,000 bp. Finally, the concentration and fragment size of the sequencing library were examined with the Qubit^®^ Fluorometer, Qubit™ dsDNA HS, and BR Assay Kits, and the TapeStation D5000 HS ScreenTape, respectively. The resultant library was sequenced on the Illumina HiSeq-PE150 platform (Table 1).

### Genome assembly and gene model prediction

*De novo* genome assembly was performed using Hifiasm (RRID:SCR_021069) [14]. Haplotypic duplications were identified and removed using purge_dups (RRID:SCR_021173) based on the depth of the HiFi reads [15]. Proximity ligation data from the Omni-C library were used to scaffold the genome assembly by YaHS [16]. Transposable elements (TEs) were annotated as previously described [17] using the automated Earl Grey TE annotation pipeline (version 1.2, https://github.com/TobyBaril/EarlGrey). A total of 38,780 papilionidae reference protein sequences were downloaded from NCBI as protein hits to perform genome annotation using Braker (v3.0.8; RRID:SCR_018964) [18] with default parameters.

## Data validation and quality control

During DNA extraction and PacBio library preparation, the samples were subjected to quality control with NanoDrop™ One/OneC Microvolume UV–Vis Spectrophotometer, Qubit^®^ Fluorometer, and overnight pulse-field gel electrophoresis. The Omni-C library was inspected by Qubit^®^ Fluorometer and TapeStation D5000 HS ScreenTape.

**Figure 2.**
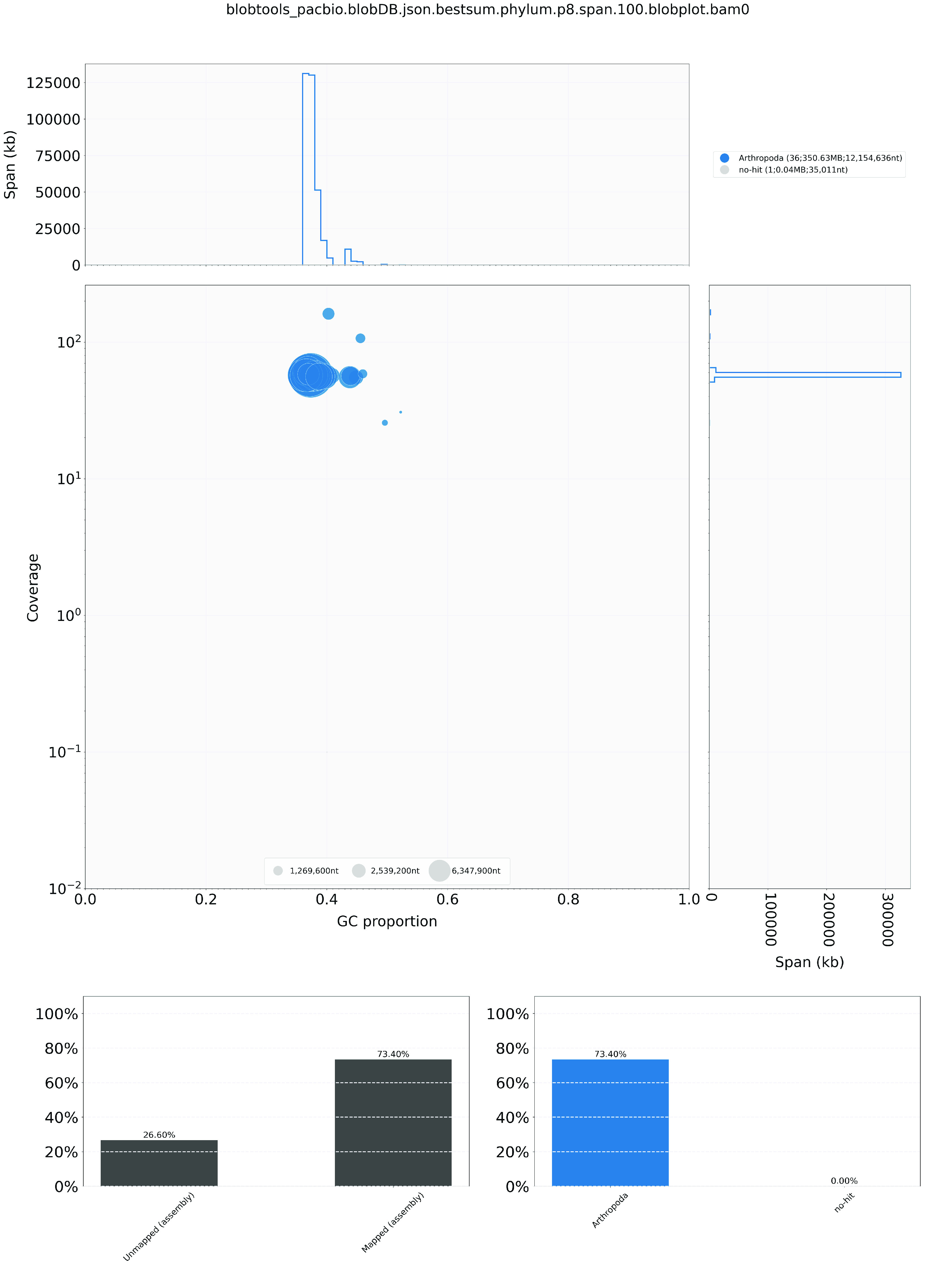
Genome assembly quality control and contaminant detection.

Regarding the genome assembly, the Hifiasm output was blast to the NT database, and the resultant output was used as input for Blobtools (v1.1.1; RRID:SCR_017618) [19]. Scaffolds that were identified as possible contamination were removed from the assembly manually (Figure 2). A statistical kmer-based approach was applied to estimate the heterozygosity of the assembled genome heterozygosity. The repeat content and the corresponding sizes were analysed using Jellyfish (RRID:SCR_005491) [20] and GenomeScope (RRID:SCR_017014) [21] (Figure 1D; Table 4). Furthermore, telomeric repeats were inspected by FindTelomeres [22]. BUSCO (v5.5.0) [23] was used to assess the completeness of the genome assembly and gene annotation with the metazoan dataset (lepidoptera_odb10).

**Table 2:** Details of the genome assembly statistics.

Species name	*Troides aeacus*
total_length	350,661,970
number	36
mean_length	9,740,610
longest	14,808,706
shortest	35,011
N_count	0.0002%
Gaps	4
N50	12,212,588
N50n	13
N70	11,032,717
N70n	19
N90	8,896,329
N90n	26
BUSCOs (Genome, lepidoptera_odb10)	C:98.8%[S:98.6%,D:0.2%],F:0.1%,M:1.1%,n:5286
HiFi (X)	78
HiFi Reads	2,805,656
HiFi Bases	27,181,071,888
HiFi Q30%	2
HiFi Q20%	4
HiFi GC%	38
HiFi Ave_len	9,688
Gene models	23,068
number of protein-coding genes	24,946
BUSCOs (Proteome, lepidoptera_odb10)	C:94.7%[S:83.9%,D:10.8%],F:0.4%,M:4.9%,n:5286
total_length of protein-coding genes	9,533,860
mean_length of protein-coding genes	382

**Figure 3.**
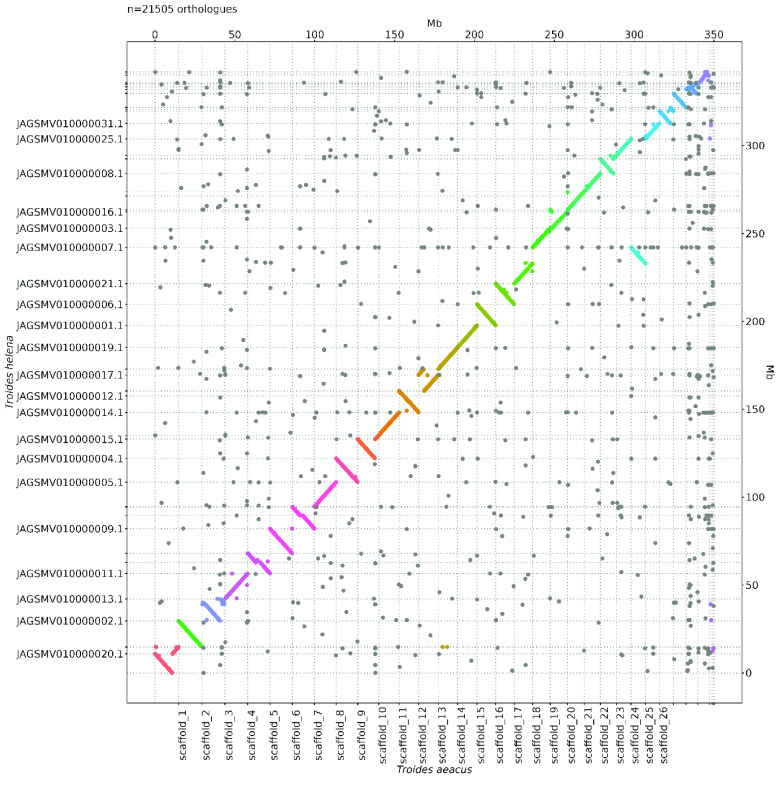
Oxford synteny plots with *Troides Helena*.

**Figure 4.**
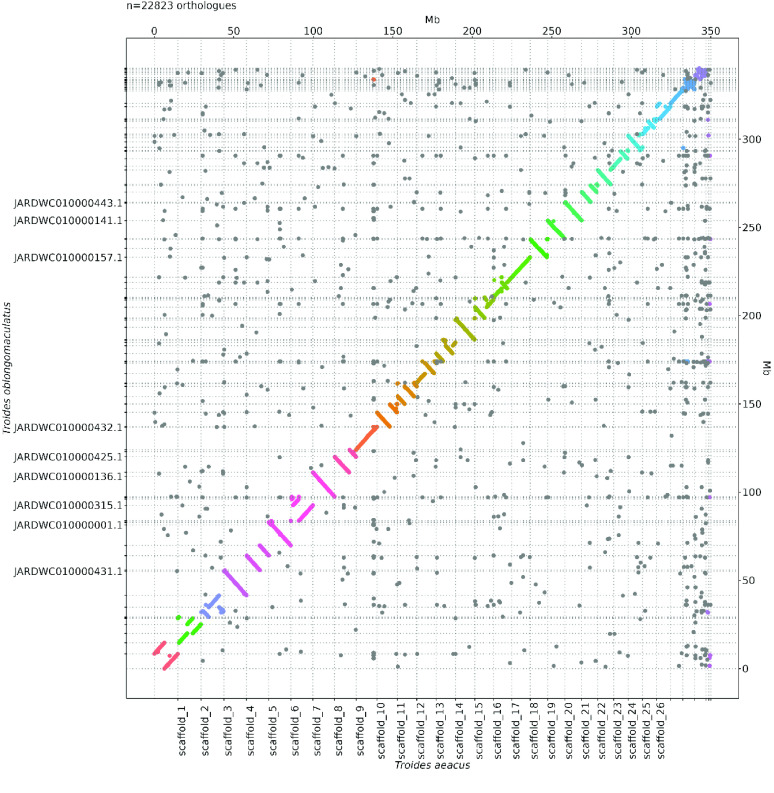
Oxford synteny plots with *Troides oblongomaculatus*.

Omni-C reads and PacBio HiFi reads were used to measure the assembly completeness and consensus quality (QV) using Merqury (v1.3; RRID:SCR_022964) [24] with kmer 19, resulting in 83.111% kmer completeness for the Omni-C data and 68.2844 QV scores for the HiFi reads, corresponding to 99.9999% accuracy. Oxford synteny plots for comparison to the same genus genome *T. helena* (GCA_029286815.1) and *T. oblongomaculatus* (GCA_029032895.1) were generated using the R package ‘ggplot2’ [25] as described in Lee *et al.* [26, 27] (Figure 3, 4 and Table 7).

**Table 3:** Information of 30 chromosomal-length scaffolds.

Chromosome no.	Scaffold name	Scaffold length (bp)	Sum of the percentage of the whole genome
1	scaffold_1	14,808,706	4.22%
2	scaffold_2	14,665,000	8.41%
3	scaffold_3	14,313,961	12.49%
4	scaffold_4	14,238,555	16.55%
5	scaffold_5	14,017,748	20.55%
6	scaffold_6	13,917,518	24.51%
7	scaffold_7	13,815,727	28.45%
8	scaffold_8	13,654,990	32.35%
9	scaffold_9	13,495,232	36.20%
10	scaffold_10	13,141,228	39.94%
11	scaffold_11	12,752,029	43.58%
12	scaffold_12	12,274,249	47.08%
13	scaffold_13	12,212,588	50.56%
14	scaffold_14	12,154,636	54.03%
15	scaffold_15	12,125,450	57.49%
16	scaffold_16	11,669,766	60.82%
17	scaffold_17	11,660,701	64.14%
18	scaffold_18	11,537,742	67.43%
19	scaffold_19	11,032,717	70.58%
20	scaffold_20	10,726,358	73.64%
21	scaffold_21	10,597,782	76.66%
22	scaffold_22	10,099,347	79.54%
23	scaffold_23	9,998,358	82.39%
24	scaffold_24	9,129,920	84.99%
25	scaffold_25	9,045,561	87.57%
26	scaffold_26	8,896,329	90.11%
27	scaffold_27	8,628,000	92.57%
28	scaffold_28	7,826,594	94.80%
29	scaffold_29	7,424,256	96.92%
30	scaffold_30	7,076,422	98.94%

**Table 4:** GenomeScope result summary (k-mer = 21).

Property	Min	Max
Homozygous (aa)	97.04%	97.11%
Heterozygous (ab)	2.89%	2.96%
Genome Haploid Length (bp)	265,767,007	268,320,884
Genome Repeat Length (bp)	69,335,920	70,002,201
Genome Unique Length (bp)	196,431,086	198,318,683
Model Fit	78.57%	99.17%
Read Error Rate	0.73%	0.73%

## Results and discussion

### Genome assembly of T. aeacus

A total of 27 Gb of HiFi bases were yielded with an average HiFi read length of 9,688 bp with 78X coverage (Supplementary Information 1). After incorporating 21.7 Gb Omni-C data, the resulting genome assembly was 350.66 Mb in size with 36 scaffolds, 30 of which are of chromosome length (Figure 1B–C; Table 2, 3). The genome has high contiguity with a scaffold N50 value of 12.21 Mb, and high completeness with a complete BUSCO (RRID:SCR_015008) estimation of 98.8% (lepidoptera_odb10) (Figure 1B, Table 2). While the genome size estimation was about 268.3 Mb with a 2.93% nucleotide heterozygosity rate (Figure 1D; Table 4), the assembled *T. aeacus* genome has a genome size similar to other swallowtail butterfly genomes, including *T. helena* (∼330 Mb) [10] and *T. oblongomaculatus* (∼348 Mb) [11]. In addition, 43 telomeres were found in 25 scaffolds of the assembly genome (Table 5). Furthermore, 23,068 gene models were predicted with a BUSCO score of 94.7% (lepidoptera_odb10).

**Table 5:** Summary of telomeric repeats found in 25 scaffolds.

Scaffold name	Strand	Position at scaffold	Sequence
scaffold_1	Forward	start	taacctaacctaacctaacctaacctaacctaacctaacctaacctaacc
scaffold_2	Reverse	end	aggttagtaggtaggttaggtaggttagttaggtaggttaggttaggtta
scaffold_4	Forward	start	aacctaacctaacctaacctaacctaacctaacctaacctaacctaacct
scaffold_4	Reverse	end	aggttaggtaggttaggttagtttaggttaggttaggttaggttaggtta
scaffold_5	Forward	start	acctaacctaacctaacctaacctaacctaacctaacctaacctaaccta
scaffold_5	Reverse	end	gttaggttaggttaggttaggttaggttaggttaggttaggttaggttag
scaffold_6	Forward	start	taacctaacctaacctaacctaacctaacctaacctaacctaacctaacc
scaffold_6	Reverse	end	aggttaggttaggttaggttaggttaggttaggttaggttaggttaggtt
scaffold_7	Forward	start	acctaacctaacctaacctaacctaacctaacctaacctaacctaaccta
scaffold_7	Reverse	end	taggttaggttaggttaggttaggttaggttaggttaggttaggttaggt
scaffold_8	Forward	start	taacctaacctaacctaacctaacctaacctaacctaacctaacctaacc
scaffold_8	Reverse	end	aggttaggttaggttaggttaggttaggttaggttaggttaggttaggtt
scaffold_9	Forward	start	ctaacctaacctaacctaacctaacctaacctaacctaacctaacctaac
scaffold_9	Reverse	end	taggttaggttaggttaggttaggttaggttaggttaggttaggttaggt
scaffold_10	Forward	start	aacctaacctaacctaacctaacctaacctaacctaacctaacctaacct
scaffold_10	Reverse	end	ttaggttaggttaggttaggttaggttaggttaggttaggttaggttagg
scaffold_11	Forward	start	cctaacctaacctaacctaacctaacctaacctaacctaacctaacctaa
scaffold_12	Forward	start	aacctaacctaacctaacctaacctaacctaacctaacctaacctaacct
scaffold_12	Reverse	end	aggttaggttaggttaggttaggttaggttaggttaggttaggttaggtt
scaffold_14	Forward	start	aacctaacctaacctaacctaacctaacctaacctaacctaacctaacct
scaffold_14	Reverse	end	aggttaggttaggttaggttaggttaggttaggttaggttaggttaggtt
scaffold_15	Forward	start	taacctaacctaacctaacctaacctaacctaacctaacctaacctaacc
scaffold_16	Reverse	end	ggttaggttaggttaggttaggttaggttaggttaggttaggttaggtta
scaffold_17	Forward	start	aacctaacctaacctaacctaacctaacctaacctaacctaacctaacct
scaffold_17	Reverse	end	ggttaggttaggttaggttaggttaggttaggttaggttaggttaggtta
scaffold_18	Forward	start	acctaacctaacctaacctaacctaacctaacctaacctaacctaaccta
scaffold_18	Reverse	end	ggttaggttaggttaggttaggttaggttaggttaggttaggttaggtta
scaffold_19	Forward	start	taacctaacctaacctaacctaacctaacctaacctaacctaacctaacc
scaffold_19	Reverse	end	aggttaggttaggttaggttaggttaggttaggttaggttaggttaggtt
scaffold_21	Forward	start	cctaacctaacctaacctaacctaacctaacctaacctaacctaacctaa
scaffold_21	Reverse	end	ttaggttaggttaggttaggttaggttaggttaggttaggttaggttagg
scaffold_22	Forward	start	taacctaacctaacctaacctaacctaacctaacctaacctaacctaacc
scaffold_22	Reverse	end	taggttaggttaggttaggttaggttaggttaggttaggttaggttaggt
scaffold_23	Forward	start	acctaacctaacctaacctaacctaacctaacctaacctaacctaaccta
scaffold_23	Reverse	end	aggttaggttaggttaggttaggttaggttaggttaggttaggttaggtt
scaffold_24	Forward	start	cctaacctaacctaacctaacctaacctaacctaacctaacctaaccaac
scaffold_24	Reverse	end	taggttaggttaggttaggttaggttaggttaggttaggttcggttaggt
scaffold_25	Forward	start	ctaacctaacctaacctaacctaacctaacctaacctaacctaacctaac
scaffold_25	Reverse	end	taggtaggtaggtaggtaggtaggtaggtaggtaggtaggtaggtaggta
scaffold_26	Reverse	end	ggttaggttaggttaggttaggttaggttaggttaggttaggttaggtta
scaffold_27	Reverse	end	taggttaggttaggttaggttaggttaggttaggttaggttaggttaggt
scaffold_28	Forward	start	taacctaacctaacctaacctaacctaacctaacctaacctaacctaacc
scaffold_28	Reverse	end	ttaggttaggttaggttaggttaggttaggttaggttaggttaggttagg

### Repeat content

A total repetitive content of 29.50% was identified in the assembled genome, including 5.16% unclassified elements (Figure 1E; Table 6). Among the known repeats, long interspersed nuclear elements (LINEs) were the most abundant ones (12.01%), followed by short interspersed nuclear element (SINE) retrotransposons (6.38%) and DNA transposons (4.71%). In contrast, Rolling Circle, long terminal repeats (LTRs), Penelope, and others were present in low proportions (Rolling Circle: 0.78%, LTR: 0.26%, Penelope: 0.20%, other: 0.02%).

**Table 6:** Summary of the repetitive elements in the genome.

Classification	Total length (bp)	Count	Proportion (%)	No. of distinct classifications
DNA	16,507,235	16,573	4.71	2,128
LINE	42,109,363	120,899	12.01	6,176
LTR	906,819	1,286	0.26	771
Other (Simple Repeat, Microsatellite, RNA)	55,850	178	0.02	86
Penelope	692,645	363	0.20	119
Rolling Circle	2,727,115	12,594	0.78	614
SINE	22,365,985	86,794	6.38	1,216
Unclassified	18,097,624	35,455	5.16	3,824
**SUM**	**103,462,636**	**274,142**	**29.50**	**14,934**

**Table 7:** Statistics of *Troides* genomes.

Species name	*Troides aeacus* (Pacbio_only_version)	*Troides aeacus*	*Troides helena*	*Troides oblongomaculatus*
total_length	350,661,170	350,661,970	346,252,535	343,353,597
number	37	36	284	457
mean_length	9,477,329	9,740,610	1,219,199	751,321
longest	25,391,358	14,808,706	20,604,617	13,649,974
shortest	35,011	35,011	4,987	526
N_count	0	800	0	4,538
Gaps	0	4	0	46
N50	12,154,636	12,212,588	11,016,300	5,909,187
N50n	12	13	13	20
N70	11,032,717	11,032,717	9,113,214	4,315,177
N70n	18	19	20	33
N90	7,826,594	8,896,329	4,177,244	1,457,309
N90n	26	26	30	55
metazoa_odb10	/	C:98.9%[S:98.3%, D:0.6%], F:0.2%, M:0.9%, n:954	C:96.6%[S:96.0%, D:0.6%], F:0.4%, M:3.0%, n:954	C:98.8%[S:98.2%, D:0.6%], F:0.3%, M:0.9%, n:954
insecta_odb10	/	C:98.9%[S:98.5%, D:0.4%], F:0.4%, M:0.7%, n:1367	C:96.7%[S:96.5%, D:0.2%], F:0.4%, M:2.9%, n:1367	C:99.1%[S:98.7%, D:0.4%], F:0.4%, M:0.5%, n:1367
lepidoptera_odb10	/	C:98.8%[S:98.6%, D:0.2%], F:0.1%, M:1.1%, n:5286	C:96.4%[S:96.2%, D:0.2%], F:0.3%, M:3.3%, n:5286	C:98.8%[S:98.6%, D:0.2%], F:0.2%, M:1.0%, n:5286
**Species**	***Troides aeacus***	***Troides helena***	***Troides oblongomaculatus***	
Number of Proteins	24,946	24,366	23,414	
Sum of Amino Acids	9,533,860	9,754,783	9,382,566	
Mean of Proteins	382	400	401	
Sum of Exons(bp)	28,601,578	29,264,202	28,147,634	
Mean of Exons	220	223	218	
Sum of Introns(bp)	92,410,884	96,334,286	93,619,605	
Mean of Introns	880	901	884	
Numer of gene loci	23,068	22,338	21,500	
Sum of gene region (bp)	105,638,137	107,935,950	105,567,879	
% of gene loci in genome	30.13%	31.17%	30.75%	
Average gene region(bp)	4,579	4,832	4,910	
metazoa_odb10	C:90.5%[S:84.6%, D:5.9%], F:1.0%, M:8.5%, n:954	C:86.2%[S:80.4%, D:5.8%], F:1.4%, M:12.4%, n:954	C:90.4%[S:84.0%, D:6.4%], F:1.3%, M:8.3%, n:954	
insecta_odb10	C:93.3%[S:82.7%, D:10.6%], F:0.4%, M:6.3%, n:1367	C:89.0%[S:78.0%, D:11.0%], F:1.1%, M:9.9%, n:1367	C:93.4%[S:82.7%, D:10.7%], F:0.6%, M:6.0%, n:1367	
lepidoptera_odb10	C:94.7%[S:83.9%, D:10.8%], F:0.4%, M:4.9%, n:5286	C:90.9%[S:79.6%, D:11.3%], F:0.8%, M:8.3%, n:5286	C:94.9%[S:83.8%, D:11.1%], F:0.5%, M:4.6%, n:5286	

## Conclusion and reuse potential

This study presents the first chromosomal-level genome assembly of the golden birdwing *T. aeacus*, a useful and precious resource for further phylogenomic studies of birdwing butterfly species in terms of species diversification and conservation.

## Data Availability

The final genome assembly was submitted to NCBI under the accession number (GCA_033220335.2). The raw reads yielded from this study were deposited on the NCBI database under the BioProject accession number PRJNA973839. The genome annotation files were deposited in figshare [28].

## Abbreviations

PBS, phosphate-buffered saline; LINE: long interspersed nuclear element; LTR: long terminal repeat; QV: consensus quality; SINE: short interspersed nuclear element; TE: transposable element.
